# Plasma levels of MMP-7 and TIMP-1 in laboratory diagnostics and differentiation of selected histological types of epithelial ovarian cancers

**DOI:** 10.1186/s13048-017-0338-z

**Published:** 2017-06-29

**Authors:** Grażyna Ewa Będkowska, Ewa Gacuta, Monika Zajkowska, Edyta Katarzyna Głażewska, Joanna Osada, Maciej Szmitkowski, Lech Chrostek, Milena Dąbrowska, Sławomir Ławicki

**Affiliations:** 1 Department of Haematological Diagnostics, Medical University Bialystok, Waszyngtona 15A, 15-269 Bialystok, Poland; 2 Department of Biochemical Diagnostics, Medical University Bialystok, Bialystok, Poland; 3 Department of Perinatology, Medical University Bialystok, Bialystok, Poland; 4 Department of Esthetic Medicine, Medical University Bialystok, Bialystok, Poland

**Keywords:** MMP-7, TIMP-1, HE4, CA125, Epithelial ovarian cancer, Tumor markers

## Abstract

**Background:**

MMP-7 and TIMP-1 may play a role in the pathogenesis of cancer disease. In this study we investigated plasma levels of selected metalloproteinase and its tissue inhibitor in comparison to plasma levels of the commonly accepted tumor markers (CA 125 and HE4) in selected histological types of epithelial ovarian cancer patients as compared to control groups: patients with a benign ovarian tumor and healthy subjects. Plasma levels of MMP-7 and TIMP-1 were determined using ELISA, CA 125 and HE4 – by CMIA methods.

**Results:**

Plasma levels of all biomarkers studied were significantly higher in ovarian cancer patients as compared to both control groups. MMP-7 demonstrated comparable to HE4 or CA125 values of diagnostic sensitivity (SE: 61%; 68%; 58%, respectively), specificity (SP: 95%; 95%; 98%, respectively), positive (PPV: 93%; 96%; 98%, respectively) and negative predictive values (NPV: 61%; 66%; 60%, respectively) in the groups tested. The combined use of the aforementioned biomarkers resulted in a further increase in diagnostic criteria and AUC, especially in the early stages of the disease.

**Conclusions:**

These findings suggest the usefulness of combining MMP-7 with CA 125 and HE4 in the diagnosis of epithelial ovarian cancer as a new tumor marker panel.

**Electronic supplementary material:**

The online version of this article (doi:10.1186/s13048-017-0338-z) contains supplementary material, which is available to authorized users.

## Background

Ovarian cancer (OC) is a highly lethal gynecological cancer. Approximately 23% of gynecological cancers are ovarian in origin, but 47% of all deaths from cancer of the female genital tract occur in women with cancer of this organ. Malignant tumors of the ovaries occur at all ages with variation in histological sub-type by age [1, 2]. Established risk factors for epithelial ovarian tumors include reproductive risk factors and inherited pathological mutations in the BRCA1 and BRCA2 genes [3, 4]. Initially, OC lacks clear symptoms, which prevents early diagnosis and treatment. Many potential biomarkers have been identified or used in recent years in the diagnostics of ovarian cancer patients [5, 6]. At present, CA 125 (carbohydrate antigen 125) [7] is the best-known ovarian cancer biomarker, although novel biomarkers such as HE4 (human epididymis protein 4), applicable to the diagnosis of this malignancy, have been researched recently [7, 8]. This glycoprotein belongs to a family of protease inhibitors and it is presumed to function as a is trypsin inhibitor. It is expressed in normal glandular epithelium of the reproductive tract, respiratory epithelium and distal renal tubules [9, 10]. In benign conditions, the highest HE4 concentrations have been observed in both women and men with renal failure. HE4 has been shown to be overexpressed in 93% of serous, 100% of endometrioid, and 50% of clear cell ovarian carcinomas. Similarly to other tumor markers, it is neither a strictly organ-specific nor a tumor-specific factor. Significant HE4 gene expression and strong immunoreactivity has been found in some lung, endometrial, renal, thyroid and breast carcinomas [9, 11]. When comparing the two aforementioned biomarkers it is believed that levels of HE4 are less frequently elevated in benign gynecological conditions than those of CA125 [12].

Different types of proteins, other than the commonly accepted and used tumor markers - such as cytokines (M-CSF, VEGF) [13, 14] and metalloproteinases are currently being investigated [15, 16]. Metalloproteinases have the ability to degrade extracellular matrix proteins, thereby facilitating tumor invasiveness, which also occurs through their interaction with growth factors, cytokines and proteases [17]. The tissue inhibitors of metalloproteinases (TIMPs: TIMP-1 and -2) regulate MMPs’ activation by binding as a complex [18]. MMP-7 (matrilysin) is the smallest proteinase from the entire metalloproteinases family with broad proteolytic activity against proteoglycans, elastin, laminin, collagens [19]. Overexpression and increased serum levels of this metalloproteinase have been confirmed in prostate, lung, renal, colorectal or breast malignancies [20–24]. Some clinical studies have also revealed significantly elevated TIMP-1 levels in the plasma of patients with colorectal, prostate and pancreatic cancers, which was associated with worse clinical outcomes [25–27].

The aim of this study was to determine plasma levels of metalloproteinase 7 and its tissue inhibitor 1 in comparison to HE4 and CA125 plasma levels in epithelial ovarian cancer patients and in relation to the control groups: patients with benign ovarian tumors and healthy subjects. The diagnostic criteria (sensitivity, specificity, predictive values of positive and negative test results) and the receiver-operating characteristic curve (ROC) for parameters investigated alone and in combinations were defined. Furthermore, correlations between the biomarkers tested were established.

These data may be used in the evaluation of the usefulness of MMP-7 and TIMP-1 in diagnosing ovarian cancer and in discriminating it from benign ovarian tumors.

## Methods

### Patients

Table 1 shows the tested groups. The study included 100 epithelial ovarian cancer patients (EOC) (sub-types *serous* and *endometrioid*) diagnosed by the Gynecology Group. Clinical stages and histological classification based on the criteria of the International Federation of Gynecology and Obstetrics (FIGO) and the World Health Organization (WHO) were established in all cases. Ovarian cancer histopathology was established in all cases by tissue biopsy of the tumor or post surgery from tumor cancer tissues. Patients with renal failure were excluded from our study due to very high HE4 concentration levels, undistinguishable from ovarian cancer. None of the patients had received chemo- or radiotherapy before blood sample collection.

**Table 1:** Characteristics of ovarian cancer patients and control groups: benign ovarian tumor and healthy subjects

Study group		Number of patients
Epithelial ovarian cancer patients		100 (100%)
• median age (range)		59 (46–87)
- sub-type *serous epithelial*		54 (54%)
• median age (range)		59 (46–81)
- sub-type *endometrioid epithelial*		46 (46%)
• median age (range)		59 (49–87)
Tumor stage	IA-T_1a_N_0_M_0_	5 (5%)
	IB-T_1b_N_0_M_0_	7 (7%)
	IC-T_1c_N_0_M_0_	13 (13%)
	IIA-T_2a_N_0_M_0_	8 (8%)
	IIB-T_2b_N_0_M_0_	9 (9%)
	IIC-T_2c_N_0_M_0_	8 (8%)
	IIIA-T_3a_N_0_M_0_	9 (9%)
	IIIB-T_3b_N_0_M_0_	9 (9%)
	IIIC-T_3c_N_0_M_0_	7 (7%)
	IV(metastases)	25 (25%)
Menopausal status:		
**-** postmenopausal		100 (100%)
Benign ovarian tumor patients		80 (100%)
- type *cystis serous*		40 (50%)
- type *cystis endometriosis*		40 (50%)
Median age (range)		54 (48–68)
Menopausal status:		
- postmenopausal		80 (100%)
Healthy subjects		80 (100%)
Median age (range)		55 (47–64)
Menopausal status:		
- postmenopausal		80 (100%)

Pretreatment staging procedures included physical and blood examinations, ultrasound scanning and chest X-rays. In addition, radioisotope bone scans, the examination of bone marrow aspirates, and brain and CT scans were performed where necessary.

The control groups were comprised of 80 benign ovarian tumor patients (*cystis serous* or *cystis endometrioides*) and 80 healthy volunteers. The benign ovarian tumor histopathology was established in all cases by tissue biopsy of the ovarian tumor or post surgery.

The healthy women group were also examined by a gynecologist prior to blood collection and subjects with a clinical history of endometriosis or mild gynecological conditions were excluded. Women included in the control group were volunteers without visible/perceptible changes in the adnexa and in the anamnesi. The group was examined by a gynecologist prior to blood collection and the ultrasound examination was performed in every case.

The ovarian cancer patients and the control group (benign lesions) were treated in the Department of Gynecology, University Hospital in Bialystok, Poland, in the years 2009–2014. The study was approved by the local Ethics Committee of the Medical University in Bialystok, numbers: R-I-002/314/2009 and R-I-002/262/2010 and all the patients gave their informed consent for study participation.

### Biochemical analyses

Venous blood samples were collected from each patient. Blood was collected into a heparin sodium tube, centrifuged 1000 rpm for 15 min. to obtain plasma samples and stored at −85^0^ C until assayed. Tested metalloproteinase-7 (MMP-7) and tissue inhibitor of metalloroteinase-1 (TIMP-1) were measured with the enzyme-linked immunosorbent assay (ELISA) (Quantikine Human Total MMP-7 Immunoassay, Human TIMP-1 Immunoassay, R&D systems) according to the manufacturer’s protocols. Duplicate samples were assessed for each patient.

The intra-assay coefficient of variation (CV%) of MMP-7 is reported to be 3.7% at a mean concentration of 4.58 ng/ml, SD = 0.168. The inter-assay coefficient of variation (CV%) of MMP-7 is reported to be 4.1% at a mean concentration of 4.82 ng/ml, SD = 0.198.The intra-assay coefficient of variation (CV%) of TIMP-1 – 5.0% at a mean concentration of 6.95 ng/ml, SD = 0.35. The inter-assay coefficient of variation (CV%) of TIMP-1 – 4.9% at a mean concentration of 6.90 ng/ml, SD = 0.34. The assay showed no significant cross-reactivity or interference with numerous human metalloproteinases and tissue inhibitors of metalloproteinases (TIMPs).

Plasma concentrations of CA125 and HE4 were measured by chemiluminescent microparticle immunoassay (CMIA) (Abbott, Chicago, IL, USA). The intra-assay CV for CA125 is reported to be 2.4% at a mean concentration of 43.5 U/ml, SD = 1.1. The inter-assay CV for CA125–3.9% at a mean concentration of 43.5 U/ml, SD = 1.7. The intra-assay CV for HE4–3.7% at a mean concentration of 39.0 pmol/L, SD = 1.4. The inter-assay CV for HE4–2.8% at a mean concentration of 39.0 pmol/L, SD = 1.1.

### Statistical analysis

Statistical analysis was performed using the STATISTICA 8.0 PL program. A preliminary statistical analysis (Chi-square test) revealed that the distribution of MMP-7, TIMP-1 and tumor markers levels did not follow a normal distribution. Consequently, nonparametric methods were used to compare tumor marker levels between patient groups. Comparisons between two groups were performed using the Mann-Whitney test. In the case of multiple groups, Kruskal-Wallis tests were calculated with post hoc comparisons according to the Dwass-Steele-Critchlow-Fligner method. ROC analyses were utilised in the evaluation of the diagnostic power of tumor markers. Markers were compared by assessing the significance of differences between the areas under their corresponding ROC curves. In addition, markers were compared by assessing the differences in sensitivity and specificity obtained for the optimal cut-off points. The construction of the ROC curves was performed using the GraphRoc Program for Windows.

Data were presented as median and range. Statistically significant differences were defined as comparisons resulting in *p* < 0.05. The Spearman rank correlation was used in the correlation analyses.

The *cut-off* of MMP-7 (5.04 ng/ml), TIMP-1 (253.33 ng/ml), HE4 (93.81 pmol/L) and CA125 (107.09 U/ml) were calculated as the 95th percentile from the control group of healthy blood donors.

## Results

In the total group of ovarian cancer (OC) patients, plasma levels of MMP-7 (5.60 ng/ml), TIMP-1 (170.79 ng/ml) and tumor markers, HE4 (207.09 pmol/L) or CA125 (139.70 U/ml) were found to be statistically higher compared to the healthy subjects (3.25 ng/ml; 128.88 ng/ml; 54.00 pmol/L; 12.70 U/ml) (*p* < 0.001, respectively) (Table 2). Moreover, we observed significant differences between the concentrations of all the parameters when every stage of cancer advancement (I-IV) was compared with the corresponding control group (with the exception of TIMP-1 – stage II): I -*p* < 0.001 (MMP-7, HE4 and CA125); II - *p* < 0.001 (in all cases); III - *p* < 0.001 (MMP-7, HE4 and CA125) and *p* = 0.001 (TIMP-1); IV - *p* < 0.001 (MMP-7, HE4 and CA125) and *p* = 0.011 (TIMP-1). Plasma concentrations of all aforementioned biomarkers were also significantly different in the advanced stages (III-IV) in comparison to those found in the early stages (I-II): MMP-7, TIMP-1, HE4 and CA125 in the comparison of stage III with stage I (*p* = 0.037; *p* = 0.005; *p* = 0.011; *p* = 0.002, respectively) and HE4 and CA125 in the comparison of stage III with stage II (*p* = 0.004; *p* = 0.013) or MMP-7, TIMP-1 and CA125 in the comparison of stage IV with I (*p* = 0.011; *p* = 0.033; *p* = 0.007), MMP-7 and CA125 in the comparison of stage IV with II (*p* = 0.010; *p* = 0.025) of tumor advancement.

**Table 2:** Plasma levels of MMP-7, TIMP-1, HE4 and CA 125 in tested groups (statistically significant when *p* < 0.05)

Groups		MMP-7 (ng/ml)	TIMP-1 (ng/ml)	HE4 (pmol/L)	CA125 (U/ml)
Ovarian cancer Median Range	Stage I	^a, b^4.76 1.98–17.86	^a^108.35 4.60–328.90	^a, b^118.70 34.50–1093.80	^a^66.73 10.60–557.20
	Stage II	^a, b^4.73 2.24–18.00	151.70 15.20–839.00	^a, b^120.90 38.30–1205.70	^a, b^61.45 9.80–2060.78
	Stage III	^a, b, d^7.92 1.98–17.80	^a, b, d^241.20 26.00–554.70	^a, b, d^650.55 48.70–1810.60	^a, b, d^766.20 10.10–2742.00
	Stage IV	^a, b, d^12.27 2.00–27.40	^a, b, d^252.60 28.00–642.00	^a, b^372.95 37.80–1944.20	^a, b, d^541.13 14.30–8602.30
	Total group	^a, b^5.60 1.98–27.40	^a, b^170.79 4.60–839.00	^a, b^207.09 34.50–1944.20	^a, b^139.70 9.80–8602.30
Control groups Median Range	Benign ovarian tumor total group	3.18 1.33–24.25	107.00 6.71–309.06	57.70 34.90–202.90	^c^22.90 5.80–748.00
	Healthy subjects	3.25 1.75–8.42	128.88 23.38–266.09	54.00 15.00–408.89	12.70 1.49–36.60

^a^Statistically significant when comparing EOC patients with healthy subjects

^b^Statistically significant when comparing EOC patients with benign ovarian tumor total group

^c^Statistically significant when comparing patients with benign ovarian tumor and healthy subjects

^d^Statistically significant when comparing EOC patients in stage III or IV with stage I or II

Ovarian cancer patients (total group) had statistically considerably higher levels of all the researched factors (*p* < 0.001; in all cases) than patients with ovarian cysts (Table 2). We also observed similar, significantly higher concentrations of MMP-7 in stages I-IV (*p* < 0.001 in all cases), of TIMP-1 in stages III-IV (*p* < 0.001), of HE4 in stages I-IV (*p* < 0.001 in all cases) and of CA125 in stages II-IV (*p* = 0.002; *p* < 0.001; *p* < 0.001) of OC in comparison with the total benign lesions group.

We also noticed significant differences in the concentrations of CA125 when the ovarian cysts group was compared with the healthy subjects group (*p* < 0.001).

Table 3 presents the diagnostic criteria of parameters tested in OC patients. We observed higher SE ranges of MMP-7 and tumor markers in more advanced ovarian tumor stages (exception – TIMP-1). They were the highest for HE4. Interestingly, MMP-7 presented better results than CA125 in the groups with stage I-II (Table 3). Combined use of the studied biomarkers resulted in an increase in diagnostic SE to a very high range in stage I: 75% and II: 81%for the combination of MMP-7 with HE4 and CA125. The maximum ranges (96–100%) were obtained for the combinations of MMP-7 + HE4; HE4 + CA125; MMP-7 + CA125 as well as for the combination of MMP-7 or its tissue inhibitor with both markers in stages III-IV (Table 4).

**Table 3:** The diagnostic criteria of MMP-7, TIMP-1, HE4 and CA 125 in epithelial ovarian cancer patients

Epithelial ovarian cancer	Diagnostic criteria (%)	MMP-7	TIMP-1	HE4	CA125
Stage I	SE	42	4	54	35
	SP	95	95	95	98
	PPV	78	25	81	90
	NPV	83	73	80	75
Stage II	SE	46	23	54	38
	SP	95	95	95	98
	PPV	78	67	82	90
	NPV	81	76	71	82
Stage III	SE	77	35	75	77
	SP	95	95	95	98
	PPV	87	75	88	95
	NPV	92	78	86	90
Stage IV	SE	79	16	88	83
	SP	95	95	95	98
	PPV	86	80	86	95
	NPV	93	76	90	92
Total group	SE	61	20	68	58
	SP	95	95	95	98
	PPV	93	87	96	98
	NPV	61	44	66	60

**Table 4:** The diagnostic criteria of MMP-7, TIMP-1 in combination with HE4 and CA 125 in epithelial ovarian cancer patients

Epithelial ovarian cancer	Diagnostic criteria (%)	MMP-7 + HE4	MMP-7 + CA125	TIMP-1 + HE4	TIMP-1 + CA 125	HE4 + CA 125	MMP-7 + HE4 + CA125	TIMP-1 + HE4 + CA 125
Stage I	SE	71	63	54	42	63	75	63
	SP	91	94	91	94	94	89	89
	PPV	74	79	68	71	79	72	68
	NPV	89	87	84	81	87	91	86
Stage II	SE	62	65	62	42	73	81	73
	SP	91	94	91	94	94	89	89
	PPV	73	81	73	73	83	75	73
	NPV	85	87	85	80	90	92	89
Stage III	SE	96	92	88	85	96	100	100
	SP	91	94	91	94	94	89	89
	PPV	81	86	79	85	86	79	79
	NPV	98	97	95	94	98	100	100
Stage IV	SE	92	96	79	83	96	96	96
	SP	91	94	91	94	94	89	89
	PPV	79	85	76	83	85	77	77
	NPV	97	98	92	94	98	98	98
Total group	SE	80	81	71	63	82	88	83
	SP	91	94	91	94	94	89	89
	PPV	93	95	92	94	95	93	92
	NPV	75	74	67	62	77	83	77

The diagnostic specificities of the biomarkers tested (SP) presented high, comparable values: 95%–98% (Table 3).

PPV in the total group of OC patients had very high values (87%–98%) for all the parameters tested, NPV was the highest for HE4 (66%) (Table 3). Combined use of the biomarkers studied for the remaining group resulted in an increase in NPV (83%) values and a decrease in PPV (95%) (Table 4). A maximum range of NPV (98–100%) was obtained for the combination of MMP-7 with HE4 and/or CA125 in stages III-IV of ovarian cancer.

To evaluate the dependence between the investigated parameters we used the Spearman’s rank correlation (Table 5). There were only positive significant correlations in the ovarian cancer total group: between the HE4 and CA125 concentrations (*R* = 0.39; *p* < 0.001), between the CA125 and MMP-7 concentrations (*R* = 0.27; *p* = 0.007,) between the CA125 and TIMP-1 concentrations (*R* = 0.30; *p* = 0.002), between the HE4 and MMP-7 concentrations (*R* = 0.35; *p* < 0.001), and between the HE4 and TIMP-1 or the MMP-7 and TIMP-1 concentrations (*R* = 0.24; *p* = 0.014). Furthermore, significant positive correlations were noticed between the HE4 and MMP-7 (*R* = 0.24; *p* = 0.008) or TIMP-1 (*R* = 0.27; *p* = 0.002) concentrations as well as between the MMP-7 and TIMP-1 concentrations (*R* = 0.25; *p* = 0.006) in the ovarian cysts group.

**Table 5:** The Spearman rank correlation for MMP-7, TIMP-1, HE4 and CA125 in tested groups

			MMP-7	TIMP-1	HE4	CA125
EOC	MMP-7	R	1.00	0.24	0.35	0.27
		p		**0.014**	**<0.001**	**0.007**
	TIMP-1	R	0.24	1.00	0.24	0.30
		p	**0.014**		**0.014**	**0.002**
	HE4	R	0.35	0.24	1.00	0.39
		p	**<0.001**	**0.014**		**<0.001**
	CA125	R	0.27	0.30	0.39	1.00
		p	**0.007**	**0.002**	**<0.001**	
Benign Ovarian Tumor	MMP-7	R	1.00	0.25	0.24	0.06
		p		**0.006**	**0.008**	0.526
	TIMP-1	R	0.25	1.00	0.27	0.001
		p	**0.006**		**0.002**	0.966
	HE4	R	0.24	0.27	1.00	0.17
		p	**0.008**	**0.002**		0.070
	CA125	R	0.06	0.001	0.17	1.00
		p	0.526	0.966	0.070	
Healthy Subjects	MMP-7	R	1.00	0.12	−0.12	−0.05
		p		0.336	0.331	0.682
	TIMP-1	R	0.12	1.00	0.13	−0.03
		p	0.336		0.286	0.794
	HE4	R	−0.12	0.13	1.00	0.14
		p	0.331	0.286		0.268
	CA125	R	−0.05	−0.03	0.14	1.00
		p	0.682	0.794	0.268	

Bold data are statistically significant when *p* < 0.05

**Fig. 1:**
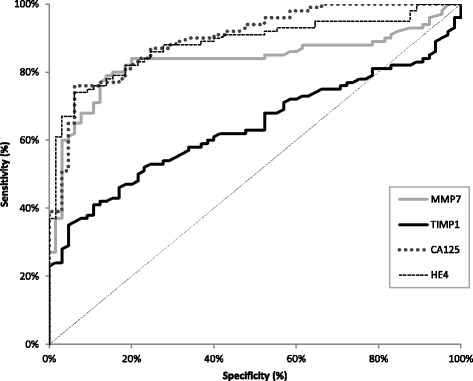
Diagnostic criteria of ROC curve for tested parameters in total EOC group

The relationship between diagnostic SE and SP was illustrated by the ROC (receiver-operating characteristics) curve. The AUCs of all compared biomarkers (with the exception of TIMP-1) were significantly higher compared to AUC = 0.5 in every studied OC group (Tables 6, 7). We demonstrated that the CA125 (0.8988) and HE4 (0.8836) areas under the ROC curve were the largest in the total group of OC (Table 6; Fig. 1). The AUCs of CA125 and HE4 were also the largest in the groups of patients with stages I-IV of the disease. Combining the studied parameters resulted in a further increase in the area under the ROC curve in every case (especially for the combination of MMP-7 + HE4 + CA125) to the value: 0.8635 in stage I; 0.9385 in stage II; 0.9935 in stage III, 0.9788 in stage IV and 0.9382 in the total OC group (Table 7). It should be emphasised that the areas under the ROC curve in various stages of cancer for MMP-7 in combination with HE4 or CA125 were as large as those for the combination of CA125 and HE4.

**Table 6:** The diagnostic criteria of the ROC curve for MMP-7, TIMP-1, HE4 and CA125 in epithelial ovarian cancer patients

Epithelial ovarian cancer	The ROC criteria	MMP-7	TIMP-1	HE4	CA 125
Stage I	AUC	0.7801^a^	0.5769	0.8343^a^	0.8324^a^
	SE	0.0693	0.0718	0.0582	0.0471
	95% C.I.	0.644–0.916	0.436–0.718	0.720–0.948	0.740–0.925
	*p*AUC = 0.5	**0.0001**	0.2840	**<0.001**	**<0.001**
Stage II	AUC	0.7938^a^	0.6109	0.8462^a^	0.8825^a^
	SE	0.0639	0.0772	0.0479	0.0397
	95% C.I.	0.669–0.919	0.460–0.762	0.752–0.940	0.805–0.960
	*p*AUC = 0.5	**<0.001**	0.1506	**<0.001**	**<0.001**
Stage III	AUC	0.8905^a^	0.7740^a^	0.9521^a^	0.9331^a^
	SE	0.0485	0.0643	0.0288	0.0327
	95% C.I.	0.796–0.986	0.648–0.900	0.896–1.008	0.869–0.997
	*p*AUC = 0.5	**<0.001**	**<0.001**	**<0.001**	**<0.001**
Stage IV	AUC	0.8679^a^	0.7314^a^	0.8994^a^	0.9458^a^
	SE	0.0624	0.0778	0.0511	0.0252
	95% C.I.	0.746–0.990	0.579–0.884	0.799–1.000	0.896–0.995
	*p*AUC = 0.5	**<0.001**	**0.0029**	**<0.001**	**<0.001**
Total group	AUC	0.8335^a^	0.6372^a^	0.8836^a^	0.8988^a^
	SE	0.330	0.0425	0.0263	0.0235
	95% C.I.	0.769–0.898	0.554–0.721	0.832–0.935	0.853–0.945
	*p*AUC = 0.5	**<0.001**	**0.0013**	**<0.001**	**<0.001**

*C.I.* – confidence intervals of AUC

^a^Statistically significant when comparing tested parameters AUC’s with 0.5 AUC

Bold data are statistically significant when *p* < 0.05

**Table 7:** The diagnostic criteria of the ROC curve for MMP-7, TIMP-1in combination with HE4 and CA125 in epithelial ovarian cancer patients

EOC	The ROC criteria	MMP-7 + HE4	MMP-7 + CA 125	TIMP-1 + HE4	TIMP-1 + CA 125	HE4 + CA 125	MMP-7 + HE4 + CA 125	TIMP-1 + HE4 + CA 125
Stage I	AUC	0.8596^a^	0.7981^a^	0.7795^a^	0.6532^a^	0.8474^a^	0.8635^a^	0.8071^a^
	SE	0.0571	0.0689	0.0668	0.0730	0.0573	0.0566	0.0646
	95% C.I.	0.748–0.972	0.663–0.933	0.648–0.910	0.510–0.796	0.735–0.960	0.752–0.974	0.681–0.934
	*p*AUC = 0.5	**<0.001**	**<0.001**	**<0.001**	**0.0359**	**<0.001**	**<0.001**	**<0.001**
Stage II	AUC	0.8456^a^	0.9284^a^	0.8290^a^	0.7479^a^	0.9331^a^	0.9385^a^	0.8982^a^
	SE	0.0521	0.0262	0.0556	0.0722	0.0255	0.0234	0.0401
	95% C.I.	0.744–0.948	0.877–0.980	0.720–0.938	0.607–0.889	0.883–0.983	0.893–0.984	0.820–0.977
	*p*AUC = 0.5	**<0.001**	**<0.001**	**<0.001**	**0.006**	**<0.001**	**<0.001**	**<0.001**
Stage III	AUC	0.9864^a^	0.9704^a^	0.9657^a^	0.9349^a^	0.9893^a^	0.9935^a^	0.9923^a^
	SE	0.0085	0.0233	0.0225	0.0412	0.0070	0.0052	0.0060
	95% C.I.	0.970–1.003	0.925–1.016	0.922–1.010	0.854–1.016	0.976–1.003	0.983–1.004	0.981–1.004
	*p*AUC = 0.5	**<0.001**	**<0.001**	**<0.001**	**<0.001**	**<0.001**	**<0.001**	**<0.001**
Stage IV	AUC	0.9513^a^	0.9519^a^	0.9673^a^	0.9423^a^	0.9487^a^	0.9788^a^	0.9526^a^
	SE	0.0406	0.0399	0.0155	0.0329	0.0370	0.0119	0.0405
	95% C.I.	0.872–1.031	0.874–1.030	0.937–0.998	0.878–1.007	0.876–1.021	0.955–1.002	0.873–1.032
	*p*AUC = 0.5	**<0.001**	**<0.001**	**<0.001**	**<0.001**	**<0.001**	**<0.001**	**<0.001**
Total group	AUC	0.9109^a^	0.9137^a^	0.8858^a^	0.8205^a^	0.9309^a^	0.9382^a^	0.9202^a^
	SE	0.0237	0.0239	0.0263	0.0332	0.0205	0.0198	0.0222
	95% C.I.	0.864–0.957	0.867–0.961	0.834–0.937	0.755–0.885	0.891–0.971	0.899–0.977	0.877–0.964
	*p*AUC = 0.5	**<0.001**	**<0.001**	**<0.001**	**<0.001**	**<0.001**	**<0.001**	**<0.001**

*C.I.* – confidence intervals of AUC

^a^Statistically significant when comparing tested parameters AUC’s with 0.5 AUC

Bold data are statistically significant when *p* < 0.05

## Discussion

Enhanced activity of matrix metalloproteinases (MMPs) and their tissue inhibitors (TIMPs) has been proven to be closely associated with tumor aggressiveness, metastasis and poor prognosis [15, 26]. In this study we investigated the diagnostic usefulness of MMP-7 and TIMP-1 separately, and in combination with HE4 and CA125, which may improve the effectiveness of non-invasive diagnostics in patients with epithelial ovarian malignancies. Furthermore, we performed a comparison of the received results with the control group results (benign ovarian lesions patients and healthy subjects). Additionally, we estimated the diagnostic utility of the aforementioned parameters in correlation to the stage of cancer disease.

Our results showed significantly higher plasma concentrations of the commonly used tumor markers in every stage of advancement as well as in the total OC group in comparison to the healthy subjects group and these results are in line with our previous papers [13, 28] and with research results published by other authors [29, 30]. We found comparable results regarding MMP-7 in the ovarian cancer [31]. Moreover, the overexpression of this metalloproteinase was associated with poor survival and/or correlated with the tumor stage of various malignancies [32–35]. Our results are consistent with the results of Määttä et al. [36] who observed increased levels of TIMP-1 in the course of ovarian cancer, although the tested group was considerably smaller (22 cases) and composed of *serous*, *mucinous* and others malignant ovarian tumors. These data are also very similar to the studies of researchers who compared patients with breast cancer [37] with healthy volunteers.

In opposition to our findings, Acar et al. [31] found no significant differences in serum MMP-7 levels in patients with benign ovarian disease (only 10 cases were included) when compared to patients with malignant disease. The results reported in the available literature regarding TIMP-1 [36–38] correspond to the results of the current study and to our previous publications regarding breast cancer [39, 40]. Regardless of the menopausal status and composition of the groups compared, statistically higher concentrations of comparative tumor markers (*p* = 0.001 up to *p* < 0.0001) were observed in ovarian cancer groups in comparison with benign diseases control groups [12, 29, 30, 41]. These results correspond to our previous publications [13, 28]. Other researchers, in line with the present study, have reported a lack of statistically significant differences in serum MMP-7 concentrations between benign ovarian lesions and healthy women groups [31]. By contrast, Beeghly-Faidel et al. [42] found a significantly higher MMP-7 expression in endometrial hyperplasia in comparison with normal endometrium. We were unable to confirm our findings regarding TIMP-1 in the published literature since no reports on the subject are available. Our present observations confirm the results of our previous study, which found significantly higher concentrations of CA125 in a group of 70 postmenopausal women with benign lesions of the ovary (cysts) [43].

The Spearman’s rank correlation test revealed that the degree of correlation between the concentrations of MMP-7, TIMP-1, HE4 and CA125 was not particularly strong (R:0.24–0.39).

This may indicate that each of the markers was elevated independently of the remaining ones and supports the proposition of a combined analysis. Unfortunately, we could not compare our data regarding MMP-7 and tissue inhibitor of metalloproteinase −1 with other publications. A positive correlation between TIMP-1 and CA15–3 concentrations in a group including 100 breast cancer patients (stages I-IV) (*R* = 0.28) has also been previously revealed [39]. Some authors have demonstrated significant positive correlations between CA125 and HE4 levels in patients with ovarian malignancies (*R* = 0.54) [44, 45].

The present study demonstrated that diagnostic sensitivity was the highest for HE4, although SE of MMP-7 reached equal or even higher values than CA125, especially in stages I-II. Our results are in agreement with the published literature [29, 46]. It is worth emphasizing that we found a maximum increase in diagnostic sensitivity for the combination of MMP-7 with both tumor markers to 75% in stage I, even to 81%–100% in stages II-IV as compared with the use of either marker alone or of both comparative tumor markers together. Several studies have confirmed this observation - they found sensitivity to be greater than in either marker used alone: MMP-7, CCL18 (CC chemokine 18), CCL11 (CC chemokine 11) and CA125 in ovarian cancer (SE in the early stages 94.4%) [46]. This conclusion is also in accordance with our previous papers in which the diagnostic criteria of selected cytokines and aforementioned tumor markers were evaluated in various gynecological malignancies [47, 48]. Diagnostic specificity (SP) reached very high and equal values for all biomarkers studied and this was in accordance [40, 43] with the available literature in the course of various malignant and benign diseases.

Notably, MMP-7 revealed high and comparable values of PPV and NPV to the values presented by HE4 and CA125 in every stage of advancement and in the total OC group. In the current study, the combination of both comparative tumor markers with MMP-7 had unquestionably higher NPV value ~100%. Unfortunately, we were unable to compare the findings concerning our diagnostic panel with the papers published since no reports on the subject are available. Interestingly, the presented results of the classic tumor markers diagnostic criteria in OC are partially in accordance with a publication by Hamed et al. [29] who observed higher values of PPV and NPV for HE4 or CA125 separately (93.1%/80.7% and 92.7%/87.2%, respectively) in 30 patients with epithelial ovarian cancer versus 20 healthy women of varying menopausal status.

The area under the ROC curve (AUC) of 1 indicates a desirable, high diagnostic power of a test. Following our analysis, HE4 (0.8836) and CA125 (0.8988) showed the largest areas under the ROC curve in the total group of ovarian cancer as well as in the groups divided according to tumor stage. Moreover, we demonstrated that the utilisation of a combined panel of MMP-7 with both known tumor markers undoubtedly improved cancer detection in every stage, but especially in the early stages of the disease (0.8343 and 0.8324 vs 0.8635; respectively – I stage). In line with the present data, preoperative serum TIMP-1 concentration showed insufficient diagnostic power (AUC = 0.730) in differentiating between low malignant potential and malignant ovarian tumors [36]. In a few previous publications the AUC values for differentiating ovarian cancer were significantly higher for the combination of various biomarkers [36, 49], which is in line with our findings [13, 28, 43]. Differences in study results might be due to differences in the histological types or disease stages of ovarian cancer and in the number of patients enrolled in each study.

## Conclusions

In summary, to the authors’ knowledge, our report is the first to evaluate the diagnostic usefulness of MMP-7 and TIMP-1 independently and, especially, in combination with both established ovarian tumor markers. The results of this study suggest that combining MMP-7, HE4 and CA125 measurements might enable the improved, early detection of selected histological types of EOC when compared with the use of either marker alone. Moreover, the investigated metalloproteinase presented similar to HE4 and CA125 diagnostic usefulness in opposition to TIMP-1 whose presented diagnostic usefulness was undoubtedly insufficient.

## Additional file

Additional file 1: Spearman rank correlation. (XLSX 10 kb)

## Abbreviations

AFP: α-fetoprotein
AUC: Area under ROC curve
CA 125: Carbohydrate antigen 125
CA19–9: Carbohydrate antigen 19–9
CCL: CC chemokine
CEA: Carcinoembryonic antigen
CMIA: Chemiluminescent microparticle immunoassay
CRP: C Reactive Protein
CT: Computed tomography
CV: Coefficient of variation
ELISA: Enzyme-linked immunosorbent assay
EOC: Epithelial ovarian cancer
FIGO: International Federation of Gynecology and Obstetrics
HE4: Human epididymis protein 4
HGFs: Hematopoietic growth factors
M-CSF: Macrophage-colony stimulating factor
MMP-7: Metalloproteinase 7
NPV: Negative predictive value
OC: Ovarian cancer
PPV: Positive predictive value
ROC: Receiver-operating characteristics
SAA: Serum amyloid A
SD: Standard deviation
SE: Diagnostic sensitivity
SP: Diagnostic specificity
TIMP-1: Tissue inhibitor of metalloproteinase-1
TNM: Tumor-nodulus-metastasis classification
VEGF: Vascular endothelial growth factor
WHO: World Health Organization
βFGF: Basic fibroblast growth factor
